# Workplace Violence Against Healthcare Professionals in Iran: A Tragic Wake-Up Call

**DOI:** 10.34172/aim.33548

**Published:** 2025-03-01

**Authors:** Maryam Modabber, Ehsan Shamsi Gooshki

**Affiliations:** ^1^Medical Ethics and History of Medicine Research Center, Tehran University of Medical Sciences, Tehran, Iran; ^2^Monash Bioethics Center, Monash University, Melbourne, Australia; ^3^Medical Ethics and History Research Center, Tehran University of Medical Sciences, Tehran, Iran

**Figure 1:**
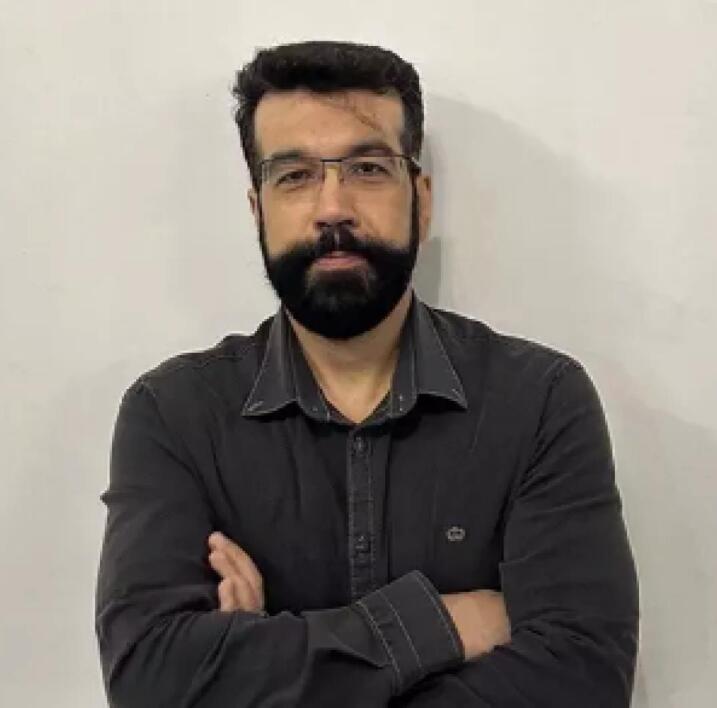
Dr. Masoud Davoodi, a cardiologist in the city of Yasuj, was murdered by the brother of a deceased patient

 During the violent murder of a cardiologist with firearms and knife, the perpetrator claimed to be seeking revenge for his brother, who died after a heart attack in a hospital in Yasuj, a city in southwestern Iran. The murderer alleged that his brother’s death was the result of a medical error by a team led by Dr. Masoud Davoodi (Figure 1). However, no error was found during a three-year investigation into the deceased patient’s family lawsuit. The public display of weapons on November 11, 2024, by the murderer has left the medical community and the general public in shock and has led to several significant responses, particularly from the Medical Council, healthcare providers (HCPs), and professional associations. Additionally, doctors in the province went on strike, halting services in non-emergency departments, which prompted one local health authority to call on higher authorities to compel doctors to return to work. Furthermore, a campaign titled “No to Violence against the Medical Community” has been initiated on social media, alongside expressions of solidarity from many professionals sharing their experiences of workplace violence (WPV).

 In recent years, WPV against HCPs has raised widespread concerns globally, becoming a public health problem that seriously endangers the health of medical staff and undermines the healthcare setting.^1^ According to the US Bureau of Labor Statistics, healthcare professionals face a 16-fold higher risk of violence compared to workers in other sectors, and they are four times more likely to need time off work due to violence.^2^ In US general hospitals, WPV represents a significant occupational risk, as indicated by reports from the Bureau of Labor Statistics. These reports highlight that between 2011 and 2013, nearly 75% of the 24 000 annual workplace assaults occurred in the healthcare sector.^3^ Worldwide, India is recognized as the “leading country” for violence against physicians, with statistics showing that as many as 75% of doctors encounter some type of violence during their careers.^4,5^ In Turkey, more than 70% of specialty physicians reported experiencing some form of violence.^6^ A study conducted in Germany found that approximately 90% of 831 doctors reported encountering some form of aggression throughout their careers, with around 70% experiencing such incidents in the past year.^5,7^ A 30-year review of workplace homicides involving Italian doctors identified 21 cases; in approximately half of these instances, the perpetrators had no documented mental disorders.^8^

 Although the prevalence of violence against healthcare workers has been identified as a significant health priority by the World Health Organization, the International Council of Nurses, and Public Services International,^9^ a shocking finding from international studies indicates that aggressive behavior towards doctors is often considered a common occurrence.^7^ Reported cases of violence represent merely the tip of the iceberg.^10^

 Despite the HCPs’ Charter of Rights, approved by the Supreme Council of the Iran Medical Council,which dedicates several articles to the security and protection of HCPs against violence,^11^ reliable data on the prevalence of WPV against HCPs is lacking. However, evidence indicates that doctors and nurses in hospitals face violence with alarming frequency. In numerous hospital emergency departments, a police unit is stationed alongside hospital security, which is another sign of the prevalence of violence that necessitates the presence of armed forces in healthcare facilities.

 Maintaining the security and safety of HCPs in clinical settings is not only for their sake but is also essential for upholding patient rights and ensuring access to services. After the recent murder of an Iranian cardiologist, most reactions to this issue focus on a few primary concerns. First, there is the inefficacy and inadequacy of the existing laws and punishments to protect healthcare professionals from violence, accompanied by calls for stricter enforcement. Second, the portrayal of the medical community in a misleading and unrealistic manner, particularly by the media. While such concerns are understandable, especially regarding the role of the media, other underlying factors are less noticed.

 Despite the complex and multifactorial nature of this social phenomenon, public trust in HCPs is a fundamental factor in this case. Although HCPs have been recognized as a key social reference group by national social capital surveys,^12^ the prevalence of violence against HCPs in Iran (often perpetrated by patients’ companions) not only signifies damage to this trust but also exacerbates distrust. Factors damaging this trust include the paternalistic clinical culture in the Iranian healthcare system, where for example informed consent is not taken seriously and the concept of therapeutic privilege often overshadows truth-telling. The possible effect of HCPs behavior and that of professional organizations on the erosion of trust should be carefully examined. Receiving illegal payments, fee-splitting, and advertising cosmetic interventions on social media could be contributing factors. All of these behaviors, along with the actions of colleagues who inadvertently provoke patients or their companions against other HCPs, can be attributed to broader structural issues, such as unjust power dynamics in clinical environments.

 Additionally, there is a lack of ongoing dialogue between HCPs and the general public. For instance, in one infamous case of medical litigation regarding the death of filmmaker Abbas Kiarostami (Palme d’Or winner from the Cannes Film Festival), resulting from a possible medical error, public sentiment was highly sensitive. The resolution of the case without clear and transparent public dialogue left many questions unanswered and damaged public trust in the process of handling medical complaints. Other possible sources of policies that may indirectly undermine trust include the extensive professional migration of HCPs to cosmetic fields (resulting from structural factors extensively advertised on social media) and the involvement of HCPs in practices like kidney transplants that involve organ buying and selling, which could include exploitation, gradually weakening public trust.

 Another crucial issue is the importance of avoiding superficial approaches that focus on punitive and criminal responses to this complex social phenomenon. While punishing individuals who assault HCPs should be part of the response, it is insufficient in the absence of more in-depth interventions. The recent murder illustrates that even severe punishments, such as the expected execution of the murderer under Iranian criminal law, do not fully address the underlying issues.

 The Iranian healthcare system and medical community, which have significantly contributed to the improvement of health indicators in the country over the past decades, are themselves facing multiple crises. These crises create stress and concern that affect patient-provider relationships. Other challenges include the increasing scarcity of healthcare resources stemming from economic sanctions imposed on Iran, indirect humiliation of the medical community through governmental support for non-evidence-based traditional medicine, and an unjustified increase in medical school acceptance capacities to compensate for physician shortages—recently exacerbated by high HCP migration rates. Limited remuneration and exploitative conditions during residency training, coupled with lack of intra-professional justice, pose significant challenges.

 Legislative gaps present another layer of complexity. There has been no development of laws and regulations to address the complex needs regulating the doctor-patient relationship. For instance, there is no law in Iran regarding life-sustaining interventions such as Do-Not-Resuscitate Orders, leading to legislative gaps that negatively impact the quality of these relationships and impose an unbearable burden on professionals.

 In conclusion, we call for evidence-informed policymaking to identify the prevalence of various forms of violence, underlying factors, and possible solutions to violence against HCPs, which requires support for well-designed research studies in the context of academic freedom. Furthermore, there is an urgent need to create and implement comprehensive legal frameworks designed specifically to address violence in healthcare settings. These frameworks should clearly define and categorize different types of violence while also establishing straightforward protocols for prevention, reporting, and response. To effectively prevent violence against HCPs, it is essential to engage a diverse range of stakeholders, including legal experts, healthcare organizations, and policymakers. By collaborating with these groups to develop and implement these legal frameworks, we can ensure that all viewpoints are taken into account, leading to practical and effective solutions.
